# Racial Disparities in Pathological Complete Response Among Patients Receiving Neoadjuvant Chemotherapy for Early-Stage Breast Cancer

**DOI:** 10.1001/jamanetworkopen.2023.3329

**Published:** 2023-03-30

**Authors:** Fangyuan Zhao, Minoru Miyashita, Masaya Hattori, Toshio Yoshimatsu, Frederick Howard, Kristiyana Kaneva, Ryan Jones, Joshua S. K. Bell, Gini F. Fleming, Nora Jaskowiak, Rita Nanda, Yonglan Zheng, Dezheng Huo, Olufunmilayo I. Olopade

**Affiliations:** 1 Department of Public Health Sciences, University of Chicago, Chicago, Illinois; 2 Department of Breast and Endocrine Surgical Oncology, Tohoku University Graduate School of Medicine, Sendai, Japan; 3 Section of Hematology and Oncology, Department of Medicine, Knapp Center for Biomedical Discovery, University of Chicago, Chicago, Illinois; 4 Department of Breast Oncology, Aichi Cancer Center, Nagoya, Japan; 5 Tempus Inc, Chicago, Illinois; 6 Department of Surgery, University of Chicago, Chicago, Illinois

## Abstract

**Importance:**

Among patients with breast cancer, inconsistent findings have been published on racial disparities in achieving pathologic complete response (pCR) after neoadjuvant chemotherapy (NACT).

**Objective:**

To investigate whether racial disparities exist in achieving pCR and what factors contribute to them.

**Design, Setting, and Participants:**

Within the ongoing Chicago Multiethnic Epidemiologic Breast Cancer Cohort (ChiMEC), which consists of a prospectively ascertained cohort of patients with breast cancer, 690 patients with stage I to III breast cancer receiving NACT were identified for this single-institution study at the University of Chicago Medicine. Patients diagnosed between 2002 and 2020 (median follow-up: 5.4 years) were included; next-generation sequencing data on tumor-normal tissue pairs were available from 186 ChiMEC patients, including both primary and residual tumor samples. Statistical analysis was performed from September 2021 to September 2022.

**Exposures:**

Demographic, biological, and treatment factors that could contribute to disparities in achieving pCR.

**Main Outcomes and Measures:**

pCR was defined as the absence of invasive cancer in the breast and axillary nodes, irrespective of ductal carcinoma in situ.

**Results:**

The study included 690 patients with breast cancer, with a mean (SD) age of 50.1 (12.8) years. Among the 355 White patients, 130 (36.6%) achieved pCR compared to 77 of the 269 Black patients (28.6%; *P* = .04). Not achieving pCR was associated with significantly worse overall survival (adjusted hazard ratio, 6.10; 95% CI, 2.80-13.32). Black patients had significantly lower odds of achieving pCR compared with White patients in the hormone receptor–negative/*ERBB2*+ subtype (adjusted odds ratio, 0.30; 95% CI, 0.11-0.81). Compared with White patients with *ERBB2*+ disease, Black patients were more likely to have MAPK pathway alterations (30.0% [6 of 20] vs 4.6% [1 of 22]; *P* = .04), a potential mechanism of anti-*ERBB2* therapy resistance. Tumor mutational burden and somatic alterations in several genes (eg, *FGF4*, *FGF3, CCND1, MCL1, FAT1, ERCC3, PTEN*) were significantly different between the primary and residual tumors.

**Conclusions and Relevance:**

In this cohort study of patients with breast cancer, racial disparities in response to NACT were associated with disparities in survival and varied across different breast cancer subtypes. This study highlights the potential benefits of better understanding the biology of primary and residual tumors.

## Key Points

**Question:**

Are there racial disparities in response to neoadjuvant chemotherapy among patients with breast cancer, and what factors contribute to them?

**Findings:**

In this cohort study of 690 patients with early-stage breast cancer, Black patients with hormone receptor–negative/*ERBB2+* disease had significantly lower odds of achieving pathological complete response compared with White patients. Black patients with *ERBB2+* disease were significantly more likely to have MAPK pathway alterations than White patients found with tumor next-generation sequencing.

**Meaning:**

These findings suggest that racial disparities in response to neoadjuvant chemotherapy varied across different breast cancer subtypes and potential therapeutic targets should be further investigated.

## Teaser

This cohort study examines whether racial disparities exist in achieving pathological complete response (pCR) after neoadjuvant chemotherapy (NACT) and what factors contribute to them among patients with breast cancer.

## Introduction

Breast cancer is a heterogeneous disease and a prototype of cancer health disparity worldwide. While breast cancer mortality has been decreasing since the 1990s in the US, Canada, and many European countries, mortality rates are rising in many countries in Asia, Latin America and Africa.^1,2^ We and others have previously described overrepresentation of aggressive subtypes as well as differences in the genomic landscape of breast cancer in Black women.^3,4,5,6^ However, there is still a paucity of data in non-European ancestry groups to gain a better understanding of the biology and heterogeneity of breast cancer to optimize breast cancer therapies through the implementation of omics-informed oncology care.^7^

Biomarker-informed clinical trials in the adjuvant vs neoadjuvant setting have demonstrated benefits of neoadjuvant chemotherapy (NACT) in patients with locally advanced or inoperable breast cancers by downsizing their tumors and increasing the chances of breast-conserving surgery.^8^ Moreover, information about response to NACT can be used to determine whether to administer additional adjuvant therapy.^9^ It has also been hypothesized that early treatment of distant micro-metastases with NACT may be beneficial for breast cancer patients that have a higher metastatic risk.^10^

Many studies have demonstrated that pathologic complete response (pCR) after NACT could be an important surrogate end point of breast cancer overall survival.^11^ Of all ethnic and racial groups, Black women have the highest mortality rate from breast cancer but there have been inconsistent findings on racial disparities in achieving pCR according to breast cancer subtypes.^12,13,14,15^ Few studies have examined the granular data on patient, tumor, and treatment characteristics that may contribute to disparities in pCR rates in diverse populations, and few have examined tumor heterogeneity and compared the landscape of somatic alterations in primary and post-NACT residual tumors. Therefore, in a large prospectively ascertained cohort, this single-institution study aims to examine whether there are racial disparities in achieving pCR among different breast cancer subtypes, and further examine the granular data on the biological, clinical, and treatment characteristics that may contribute to such disparities.

## Methods

This cohort study was approved by the University of Chicago institutional review board. All participants provided their written informed consent. We followed the Strengthening the Reporting of Observational Studies in Epidemiology (STROBE) reporting guideline.^16^

### Study Population

The Chicago Multiethnic Epidemiologic Breast Cancer Cohort (ChiMEC) consists of 4591 patients with breast cancer who were prospectively ascertained and recruited at the time of diagnosis at the University of Chicago Medicine between 1993 and 2021, spanning nearly 2 decades.^17,18^ From them, we identified 690 patients (diagnosed between 2002 and 2020, median follow-up: 5.4 years) with invasive nonmetastatic (ie, stage I, II, or III) breast cancer who received NACT for this analysis (eFigure 1 in Supplement 1). The general clinical rationale for administering NACT was to shrink or downstage a locally advanced tumor to make it operable, and to monitor their response to chemotherapy to adjust postsurgery treatment; while for each individual patient the decision to do so and the regimen administered were based on the treating physician’s discretion and standard clinical practice. To examine the landscape of somatic alterations in primary and residual tumors, we sequenced tumor-normal tissue pairs in a subset of 186 patients with available tumor specimens in our biobank, including both patients who received NACT and those who received adjuvant therapy only, with a higher sampling probability given to patients whose tumors recurred (eTable 1 in Supplement 1). Among the 186 patients, 13 patients had matched primary and residual tumor samples after NACT.

### Patient Characteristics and Study Endpoints

pCR after NACT was defined as the absence of invasive cancer in the breast and axillary nodes, irrespective of ductal carcinoma in situ (ypT0/is ypN0). Demographic data including self-reported race and/or ethnicity were collected through survey at baseline and confirmed by reviewing medical records. Race and ethnicity categories included non-Hispanic Black, non-Hispanic White, and other (which included Asian, Hispanic, and American Indian or Alaska Native). Clinical data were collected from medical records and pathology reports. Based on the expression status of hormone receptors (HR) estrogen receptor (ER) and progesterone receptor (PR), and the amplification of human epidermal growth factor receptor 2 (*ERBB2* [formerly *HER2*]), patients were grouped into 4 subtypes: HR+/*ERBB2−* (ER+ and/or PR+, *ERBB2−*), HR+/*ERBB2*+ (ER+ and/or PR+, *ERBB2+*), HR-/*ERBB2+* (ER-, PR-, *ERBB2+*) and triple-negative breast cancer (TNBC; ER-, PR-, *ERBB2−*).^19^ We also extracted detailed data on the *ERBB2* to chromosome 17 (*ERBB2*/CEP17) ratio and calculated the Histo (H)-scores using the percentage and intensity of tumor cell nuclei that positively stained for ER and PR.^20^ HR+/*ERBB2−* patients were further categorized into luminal A-like and luminal B-like based on the current European Society for Medical Oncology guideline.^21^ Comorbidity was represented by the Deyo/Charlson comorbidity index (CCI)^22^ calculated at the date of diagnosis. Treatment data were extracted from medical and pharmacy records. The survival outcomes were followed through the hospital-based cancer registry, clinical visit records, and periodic searches in the National Death Index.^23^

### Molecular Profiling and Comparative Analyses

Single nucleotide variants, small insertions and deletions, copy number alterations, and chromosomal rearrangements were called by the Tempus xT assay, which includes a panel of targeted genes, as previously described.^24^ Tumor mutation burden (TMB) was calculated by dividing the number of nonsynonymous mutations by the megabase size of the panel, and a TMB greater than 9 mutations per million base pair of DNA (m/MB) was considered high.^24^ We examined the genes that were previously reported as breast cancer driver genes,^5,25^ or belonged to any of the 10 pan-cancer canonical pathways (ie, cell cycle, Hippo, Myc, Notch, Nrf2, PI-3-Kinase/Akt, RTK-RAS, TGFβ signaling, p53, and β-catenin/Wnt).^26^ We also examined 2 pathways downstream of *ERBB2* activity (PI3K/AKT and MAPK pathways) that were previously reported to result in resistance to anti-*ERBB2* therapy.^27^ The genes involved in the MAPK pathway were *EGFR*, *NF1*, *KRAS*, *BRAF*, and *MAP2K*; for the PI3K/AKT pathway they were *PIK3CA*, *PTEN*, and *AKT1*.^27^ The pathway was considered altered if any somatic alterations were observed in its component genes, and we compared the frequency of pathway alterations between Black and White patients with *ERBB2+* disease.

### Statistical Analysis

Patient characteristics were compared using *t* tests for normally distributed continuous variables, Wilcoxon Rank-sum tests for skewed continuous variables and ordinal variables, and χ^2^ tests and Fisher exact tests for categorical variables. Multivariable logistic regression was used to examine the tumor and treatment factors that contributed to the odds of achieving pCR among all cases and stratified by subtypes. Cox proportional hazards models were used to assess the association between pCR and overall and recurrence-free survival. Exact logistic regression and Fisher exact tests were used to compare differences in the somatic alterations between primary and residual diseases among all samples and stratified by subtypes. The significance threshold was *P* < .05, and the tests were all 2-sided. Statistical analyses were conducted using the Stata 16 software package (StataCorp) and R statistical software version 4.1.2 (R Project for Statistical Computing) from September 2021 to September 2022.

## Results

### Patient Characteristics

The study included 690 patients with breast cancer (stage I, II, or III) receiving NACT and mean (SD) age of 50.1 (12.8) years. At time of diagnosis, the mean (SD) age was 52.7 (13.7) years for the 269 non-Hispanic Black (Black) patients and 48.7 (11.8) years for the 355 non-Hispanic White (White) patients (Table 1). TNBC (35.1%) and HR+/*ERBB2−* (32.5%) were the most common subtypes, followed by HR+/*ERBB2*+ (20.4%) and HR-/*ERBB2*+ (12.0%). More than 75% of the patients had grade 3 disease (ie, faster-growing) in this cohort. Compared with White patients, Black patients were less likely to achieve pCR, more likely to be diagnosed at more advanced stages, had a higher comorbidity burden, experienced longer delays in chemotherapy initiation, had longer durations of chemotherapy, and were more likely to be Medicaid-covered. Adjusting for insurance type did not fully explain the racial difference in delays, with Black patients having 7.1 days (95% CI, 2.9-11.3 days; *P* < .001) longer time from diagnosis to chemotherapy than White patients.

**Table 1.** Characteristics of Patients With Invasive Nonmetastatic Breast Cancer Undergoing NACT Stratified by Race and Ethnicity

Factor	Patients, No. (%) (N = 690)			*P* value^b^
	Black (n = 269)	White (n = 355)	Others (n = 66)^a^	
pCR				
No	192 (71.4)	225 (63.4)	44 (66.7)	.04
Yes	77 (28.6)	130 (36.6)	22 (33.3)	
Age at diagnosis, mean (SD), y	52.7 (13.7)	48.7 (11.8)	46.8 (12.9)	<.001
Breast cancer subtype				
HR+/*ERBB2*−	78 (29.0)	126 (35.5)	20 (30.3)	.31
HR+/*ERBB2*+	53 (19.7)	71 (20.0)	17 (25.8)	
HR−/*ERBB2*+	34 (12.6)	42 (11.8)	7 (10.6)	
TNBC	104 (38.7)	116 (32.7)	22 (33.3)	
Tumor grade				
1	5 (2.0)	9 (2.6)	0	.39
2	47 (18.4)	77 (22.5)	22 (33.8)	
3	203 (79.6)	256 (74.9)	43 (66.2)	
Missing	14	13	1	
AJCC stage				
I	27 (10.0)	62 (17.5)	13 (19.7)	.02
II	162 (60.2)	208 (58.6)	45 (68.2)	
III	80 (29.7)	85 (23.9)	8 (12.1)	
Clinical T-stage				
T0	1 (0.4)	0	1 (1.5)	.002
T1	37 (13.8)	85 (23.9)	11 (16.7)	
T2	153 (56.9)	192 (54.1)	40 (60.6)	
T3	56 (20.8)	57 (16.1)	13 (19.7)	
T4	22 (8.2)	21 (5.9)	1 (1.5)	
Clinical N-stage				
N0	111 (41.3)	167 (47.0)	40 (60.6)	.15
N1-3	158 (58.7)	188 (53.0)	26 (39.4)	
CCI^c^				
0	208 (77.3)	318 (89.6)	59 (89.4)	<.001
1	24 (8.9)	19 (5.4)	5 (7.6)	
≥2	37 (13.8)	18 (5.1)	2 (3.0)	
Delay in chemotherapy, wk^d^				
≤4	86 (32.0)	135 (38.0)	27 (40.9)	.007
4-8	127 (47.2)	181 (51.0)	30 (45.5)	
>8	56 (20.8)	39 (11.0)	9 (13.6)	
Duration of chemotherapy, wk^e^				
≤15	66 (28.2)	91 (38.2)	19 (36.5)	.03
15-20	121 (51.7)	107 (45.0)	24 (46.2)	
>20	47 (20.1)	40 (16.8)	9 (17.3)	
Missing	35	117	14	
Insurance type				
Private insurance	129 (48.7)	290 (82.6)	48 (77.4)	<.001
Medicare	51 (19.2)	46 (13.1)	9 (14.5)	
Medicaid	85 (32.1)	15 (4.3)	5 (8.1)	
Other or unknown	4	4	4	

Abbreviations: AJCC, American Joint Committee on Cancer; CCI, Charlson Comorbidity Index; *ERBB2*−, human epidermal growth factor receptor 2–negative; *ERBB2*+, human epidermal growth factor receptor 2–positive; HR−, hormone receptor–negative; HR+, hormone receptor–positive; NACT, neoadjuvant chemotherapy; pCR, pathologic complete response; TNBC, triple-negative breast cancer.

^a^ Other patients include 35 Asian patients, 30 Hispanic patients, and 1 American Indian or Alaska Native patient.

^b^ *P* values for the comparison between White patients and Black patients were estimated using *t* tests for age at diagnosis; χ^2^ tests for pCR, tumor subtype, tumor grade (excluding missing categories), tumor stage, and CCI; Wilcoxon Rank-Sum tests for the clinical T-stage and N-stage, delay in chemotherapy and duration of chemotherapy (excluding missing categories).

^c^ The most common comorbidities in our cohort were other cancers (5.7%), type 2 diabetes (5.4%), and pulmonary diseases (3.5%).

^d^ Delay in chemotherapy initiation was defined as the time from diagnosis to receipt of the first chemotherapy.

^e^ Duration of chemotherapy was defined as the time between the first and the last date of chemotherapy in the medical records.

### Survival Analysis

**Figure 1.**
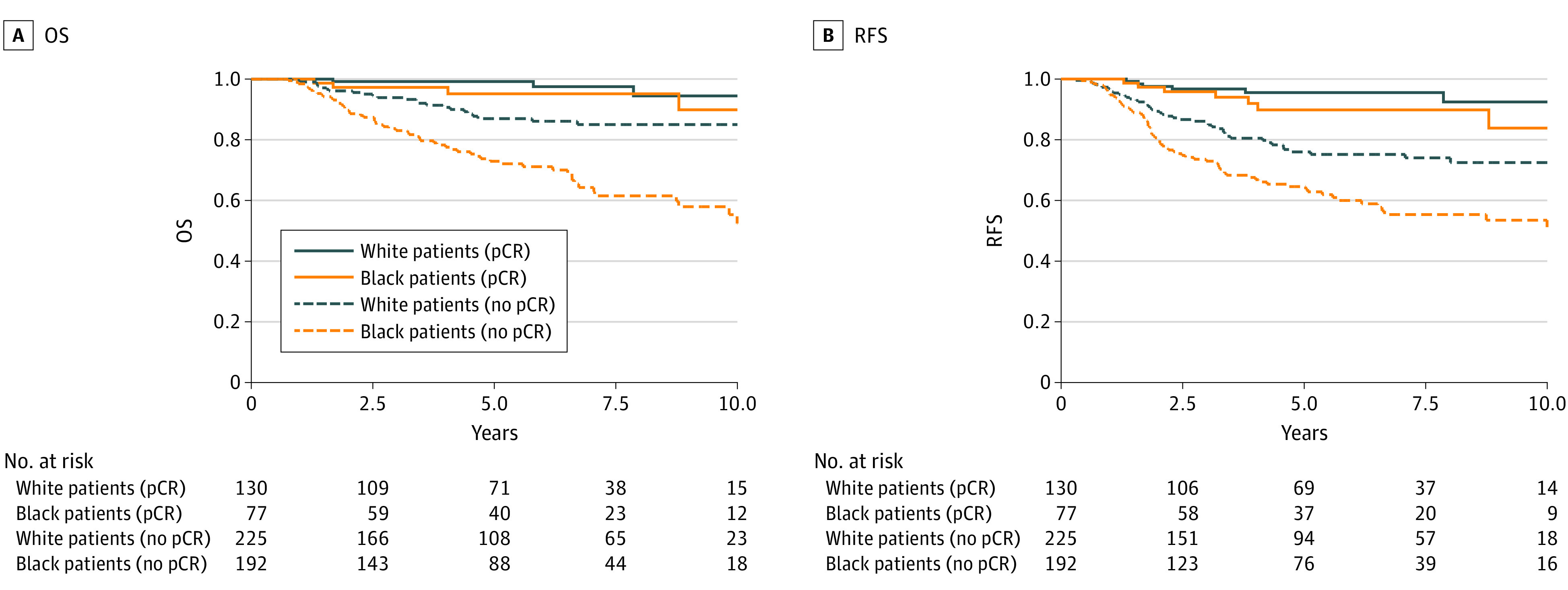
Overall Survival (OS) and Recurrence-Free Survival (RFS) Graphs Between Black and White Patients Stratified by Pathological Complete Response (pCR) Status A, OS (defined as the time from the date of diagnosis to the date of death from any cause or date of last follow-up) graphs between Black and White patients stratified by pCR status. B, RFS (defined as the time from the date of diagnosis to the first appearance of one of the following: invasive recurrence of breast cancer, death from any cause or date of last follow-up) graphs between Black and White patients stratified by pCR status.

Among the 355 White patients, 130 (36.6%) achieved pCR compared with 77 of the 269 Black patients (28.6%; *P* = .04) (Table 1). With a median (IQR) follow-up of 5.4 (2.5-7.8) years, we found that achieving pCR was significantly associated with improved survival (Figure 1). The results of the multivariable Cox regression model (eTable 2 in Supplement 1) showed that compared with those achieving pCR, patients not achieving pCR experienced an approximately 6-fold increased hazard in both overall survival (adjusted hazard ratio [aHR], 6.10 [95% CI, 2.80-13.32]) and recurrence-free survival (aHR, 5.54 [95% CI, 3.10-9.88]). Furthermore, Black patients had a 137% higher overall mortality risk than White patients (aHR, 2.37 [95% CI, 1.49-3.77]).

### Racial Disparities in pCR Among Different Subtypes

Compared with White patients, the odds ratio (OR) of achieving pCR for Black patients overall was 0.69 (95% CI, 0.49-0.98). After sequentially adjusting for selected patient, tumor, and treatment characteristics (eTable 3 in Supplement 1), the adjusted OR (aOR) of achieving pCR for Black vs White patients became statistically insignificant (aOR, 0.72 [95% CI, 0.49-1.06]). HR+/*ERBB2*- subtype, older age at diagnosis, higher clinical T-stage and N-stage, longer delay in initiation of chemotherapy, and shorter duration of chemotherapy were all significantly associated with lower odds of achieving pCR (eTable 4 in Supplement 1). There was no significant difference between Black and White patients in treatment regimen received (eTable 5 in Supplement 1).

We also examined racial differences in pCR by tumor subtype (Table 2). The racial disparity in pCR was largest among patients with HR-/*ERBB2*+ disease, where 73.8% of White patients (31 of 42) achieved pCR while the rate was only 41.2% (14 of 34) for Black patients (aOR, 0.30 [95% CI, 0.11-0.81]). Among the HR+/*ERBB2−* subtype, the pCR rate was 20.5% (16 of 78) for Black patients while it was 19.8% (25 of 126) for White patients. Among the HR+/*ERBB2+* subtype, the pCR rate was 24.5% (13 of 53) for Black patients and 38.0% (27 of 71) for White patients. The pCR rate was 32.7% (34 of 104) for Black patients with TNBC, and the rate was 40.5% (47 of 116) for White patients.

**Table 2.** Racial Disparity in the Odds of Achieving pCR Stratified by Subtype and Adjusted for Selected Tumor and Treatment Characteristics

	pCR rate, No./total No. (%)		OR (95% CI)	aOR (95% CI)	
	Black patients	White patients	Black vs White patients	Black vs White patients^a^	Black vs White patients^b^
Overall	77/269 (28.6)	130/355 (36.6)	0.69 (0.49-0.98)	0.73 (0.51-1.05)	0.81 (0.56-1.17)
Subtype					
HR+/*ERBB2*−	16/78 (20.5)	25/126 (19.8)	1.04 (0.52-2.11)	1.11 (0.54-2.26)	1.18 (0.57-2.42)
HR+/*ERBB2*+	13/53 (24.5)	27/71 (38.0)	0.53 (0.24-1.16)	0.58 (0.26-1.29)	0.63 (0.28-1.41)
HR−/*ERBB2*+	14/34 (41.2)	31/42 (73.8)	0.25 (0.09-0.65)	0.25 (0.09-0.66)	0.30 (0.11-0.81)
TNBC	34/104 (32.7)	47/116 (40.5)	0.71 (0.41-1.24)	0.95 (0.53-1.70)	1.04 (0.58-1.86)

Abbreviations: aOR, adjusted odds ratio; *ERBB2*−, human epidermal growth factor receptor 2–negative; *ERBB2*+, human epidermal growth factor receptor 2–positive; HR−, hormone receptor–negative; HR+, hormone receptor–positive; OR, odds ratio; pCR, pathologic complete response; TNBC, triple-negative breast cancer.

^a^ Adjusted for age at diagnosis, clinical T-stage, clinical N-stage and/(or stratified by) subtype.

^b^ Adjusted for age at diagnosis, clinical T-stage, clinical N-stage, delay in treatment, and/(or stratified by) subtype.

To gain a deeper understanding of the racial disparities by subtypes, we further investigated potential molecular features (ie, ER and PR H-scores, luminal A/B-like, *ERBB2*/CEP17 ratios) associated with pCR. Among patients with HR+/*ERBB2*- disease (eTable 6 in Supplement 1), we found that Black patients had substantially lower ER and PR H-scores, while decreased ER H-score and PR H-score were both significantly associated with higher odds of pCR (eTable 7 in Supplement 1). Further adjusting for ER and PR H-scores in addition to age at diagnosis, clinical T- and N-stage and delay in treatment substantially neutralized the aOR of pCR seen in Black vs White patients with HR+/*ERBB2*- disease, reducing it from 1.92 (95% CI, 0.81-4.59) to 1.37 (95% CI, 0.53-3.55). Among Black patients with HR+/*ERBB2*- disease, 74.3% (52 of 70) had luminal B-like tumors vs 61.3% (57 of 93) of White patients (*P* = .08); and patients with luminal B-like disease were more likely to achieve pCR than patients with luminal A-like disease (aOR, 3.15; 95% CI, 1.25-7.88). Similarly, further adjusting for luminal subtypes in addition to age, tumor stage, and delay in treatment can substantially neutralize the aOR of pCR between Black and White patients from 1.28 (95% CI, 0.59-2.78) to 1.10 (95% CI, 0.29-2.45). Among patients with HR-/*ERBB2+* disease, *ERBB2*/CEP17 ratio data were available for about 65% of them, and we found that Black patients had a median *ERBB2*/CEP17 ratio of 6.3 compared with 6.5 for White patients (*P* = .92).

### DNA Sequencing Results From Primary and Residual Tumor Samples

**Figure 2.**
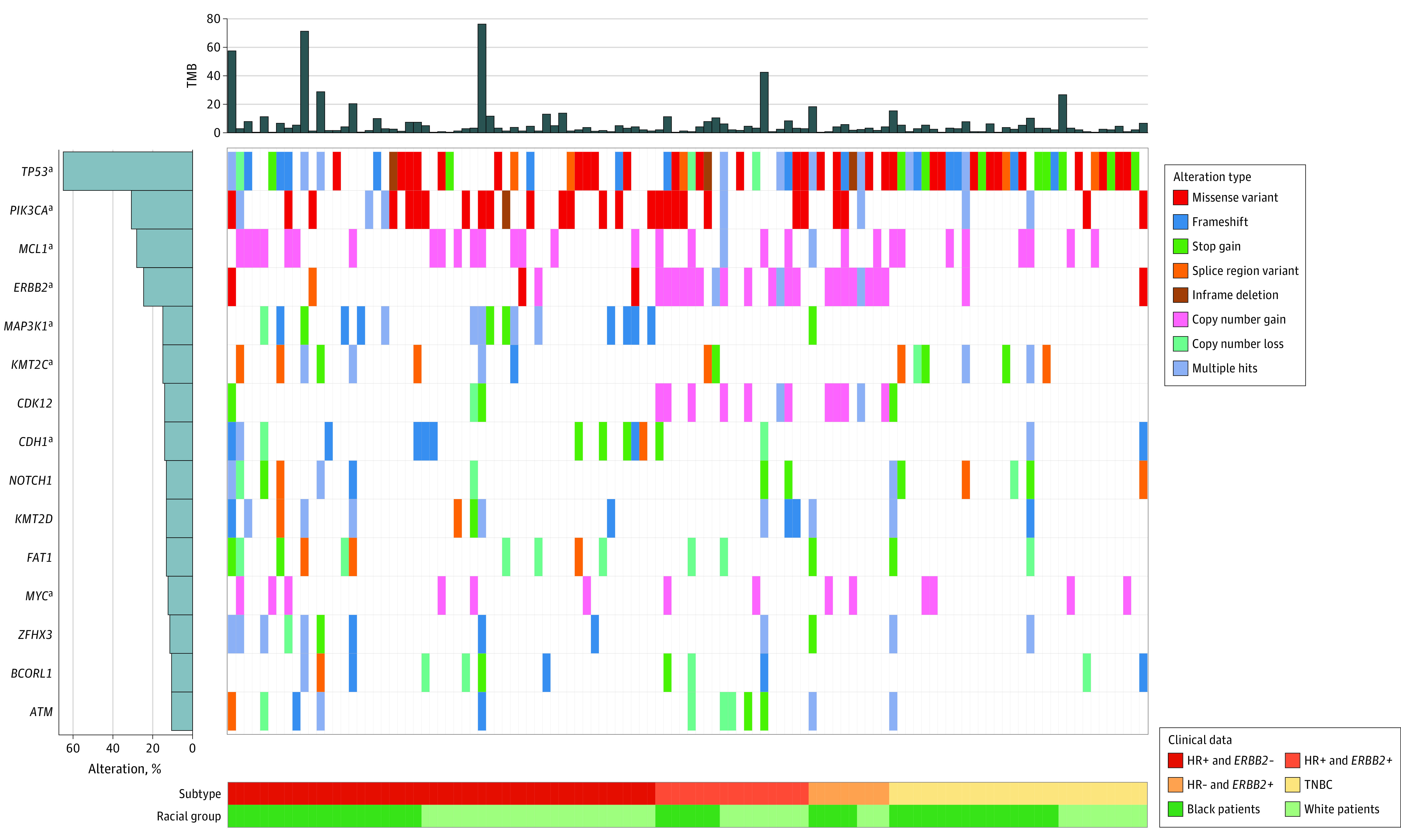
Mutational Landscape of Primary Tumor Samples From 114 Black and White Patients Stratified by Breast Cancer Subtypes The Oncoprint demonstrated the genes that had point mutations, copy number variations, and/or chromosomal variation observed for at least 10% of the samples. Genes were ordered by decreasing frequency. Not all 153 primary tumor samples were plotted. Samples were not plotted if no alterations were observed for the listed genes, the subtype data were missing and/or they were collected from patients of other racial or ethnic groups. See eFigure 3 in Supplement 1 for a more comprehensive Oncoprint. *ERBB2*− indicates human epidermal growth factor receptor 2–negative; *ERBB2+*, human epidermal growth factor receptor 2–positive; HR−, hormone receptor–negative; HR+, hormone receptor–positive; TNBC, triple-negative breast cancer. ^a^Genes that were previously reported as breast cancer driver genes.

**Figure 3.**
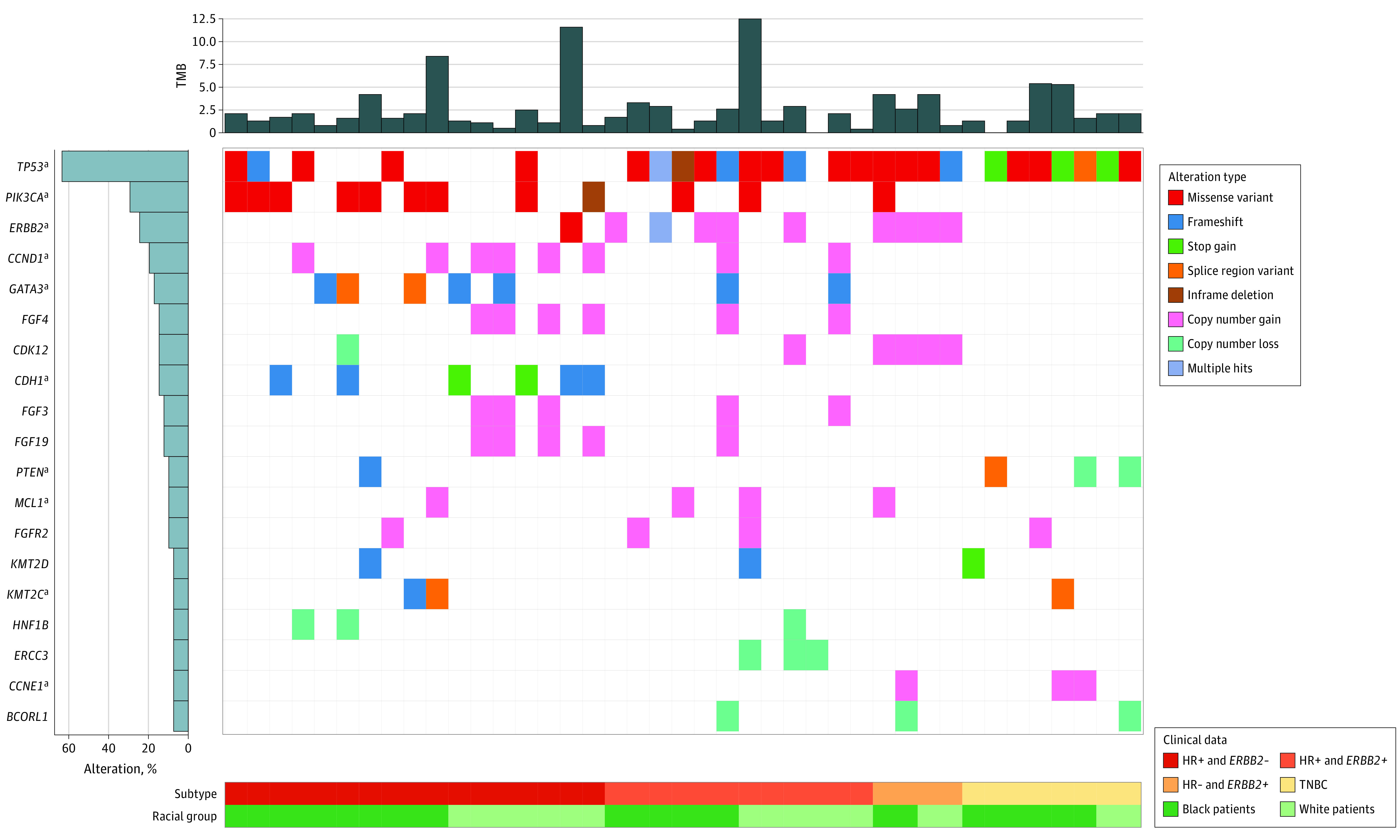
Mutational Landscape of Residual Tumor Samples From 41 Black and White Patients Stratified by Breast Cancer Subtypes The Oncoprint demonstrated the genes that had point mutations, copy number variations, and/or chromosomal variation observed for at least 7% of the samples. Genes were ordered by decreasing frequency. Not all 46 residual tumor samples were plotted. Samples were not plotted if no alterations were observed for the listed genes, the subtype data were missing and/or they were collected from patients of other racial/ethnic groups. See eFigure 4 in Supplement 1 for a more comprehensive Oncoprint. *ERBB2*− indicates human epidermal growth factor receptor 2–negative; *ERBB2+*, human epidermal growth factor receptor 2–positive; HR−, hormone receptor–negative; HR+, hormone receptor–positive; TNBC, triple-negative breast cancer. ^a^Genes that were previously reported as breast cancer driver genes.

The somatic alterations observed in the primary (Figure 2) and residual tumor samples (Figure 3) showed substantial discordance. As is shown in eTable 8 in Supplement 1, amplification of *FGF4*, *FGF3*, and *CCND1* occurred more frequently among residual tumors, whereas amplification of *MCL1* occurred more frequently among primary tumors. *FAT1* loss was only found among primary tumors. After further stratifying by subtypes, we found more *CCND1* and *FGF4* amplification among HR+/*ERBB2*- residual tumors, significantly more *ERCC3* loss among *ERBB2*+ residual tumors, and significantly more *PTEN* loss among TNBC residual tumors. We also observed a substantial loss of *ERBB2* amplification among *ERBB2*+ residual tumors. Through Wilcoxon rank-sum tests, we found that the median (IQR) TMB of primary tumors was significantly higher than that of residual tumors (3.2 [1.3-6.3] m/MB vs 1.9 [1.3-3.3] m/MB, *P* = .007), whereas there were no statistically significant racial differences in TMB observed. Among the primary tumors, 15.5% (13 of 84) of the HR+ samples had high TMB (>9 m/MB) and 7.1% (6 of 84) of the HR+ samples had TMB greater than 20 m/MB. Through examining 2 potential anti-*ERBB2* therapy resistance pathways, we found that MAPK pathway alterations occurred in 30.0% (6 of 20) of the tumors of Black patients with *ERBB2*+ disease, whereas only in 4.6% (1 of 22) of the tumors of White patients (*P* = .04). PI3K/AKT pathway alterations were observed in 45.0% (9 of 20) of the tumors of Black patients with *ERBB2*+ disease and in 18.2% (4 of 22) of the tumors of White patients, but this was not a statistically significant difference (*P* = .10).

To explore tumor evolution post-NACT in the same patient, we examined 13 patients with matched pairs of primary and residual tumor. As shown in the mutational landscape graph (eFigure 2 in Supplement 1), primary tumors and residual tumors had distinct somatic alterations. Among 6 tumors that were initially diagnosed as *ERBB2*+, 1 no longer had *ERBB2* amplification post-NACT. *CKS1B* gene was found to be amplified in 6 of 13 (45%) primary tumors but in none of the residual tumors, although the numbers are too small to make definitive conclusions. The median (IQR) TMB was 4.2 (3.2-7.4) m/MB in the primary tumors and 2.1 (1.3-4.2) m/MB in the residual tumors (*P* = .36).

## Discussion

In this single-institution prospective cohort, we found that achieving pCR was associated with improved long-term survival outcomes, and that Black patients were less likely to achieve pCR compared with their White counterparts in general. We found that longer delay in initiation of chemotherapy and shorter duration of chemotherapy were significantly associated with lower odds of achieving pCR. Previous studies in the adjuvant setting had similar findings.^28,29^ Further investigation is needed to better understand the contribution of systemic racism and institutional barriers on the treatment disparities observed. In this study, we found no significant differences between Black and White patients in the actual treatment regimens received. We observed that the racial disparity in pCR was most profound among patients with HR-/*ERBB2*+ disease. Given that pCR rate for women with HR-/*ERBB2*+ disease was also highest of all breast cancer subtypes, as shown in this and other studies,^30^ disparate response to similar treatment plans suggested the existence of underexplored biological differences.

By performing next-generation sequencing (NGS) on primary and residual tumors, this study identified that alterations in 2 pathways, MAPK and PI3K/AKT, occurred more frequently among Black patients with *ERBB2*+ disease compared with White patients. Alterations in these 2 pathways were previously reported to result in resistance to anti-*ERBB2* therapy.^27^ On the other hand, they also have the potential to serve as viable therapeutic targets to be combined with anti-*ERBB2* therapies.^31,32^ We also found that Black patients had slightly lower *ERBB2*/CEP17 ratio compared with White patients, which was previously shown to be associated with lower pCR rates.^33^ Although the sample size of this study did not provide sufficient power to detect a statistically significant difference, our analysis of the National Cancer Database (NCDB) confirmed the importance of *ERBB2*/CEP17 ratio (ie, low *ERBB2* status).^34^

To better understand the potential racial difference in pCR rate among patients with HR+/*ERBB2*- disease, we further looked at their heterogeneity in ER and PR positivity as well as stratifying them to luminal A/B-like diseases. Consistent with other studies,^35,36,37,38^ we showed that luminal B-like tumors, and those with lower ER and PR positivity, had higher odds of achieving pCR. Meanwhile, we found that 74.3% (52 of 70) of Black patients with HR+/*ERBB2*- had luminal B-like tumors (vs 61.3% [57 of 93] of White patients; *P* = .08) and had lower ER and PR H-scores than White patients, which was also consistent with previous studies.^39,40,41^ Adjusting for ER and PR H-scores (or luminal A/B) can substantially neutralize the differences in pCR between Black and White patients with HR+/*ERBB2*-, although the results were statistically insignificant limited by sample size. For patients with ER-low tumors, several ongoing clinical trials are available to test the potentials of CDK4/6 inhibitors, molecular-targeted or immunotherapy options rather than a traditional single agent approach to endocrine therapy.^42^ Through NGS, we also found that 15% of the HR+ primary tumors had high TMB, indicating that they might benefit from additional immune checkpoint blockade therapies.^43,44^

Lastly, comparing the somatic alterations between primary and post-NACT residual tumors, we found substantial loss of *ERBB2* amplification among the *ERBB2*+ residual tumor samples after NACT, which has also been seen in other studies.^45,46^ We also found decrease of *TOP2A* and *PIK3CA* alterations among *ERBB2*+ residual tumors, and a significant increase of *PTEN* loss among TNBC residual tumors, consistent with previous reports.^47,48,49^ These alterations point to alternative pathways that could be potential therapeutic targets. In addition, we observed a significant enrichment of fibroblast growth factors (FGFs) among residual tumors, which could potentially be treated with new therapies targeting FGF/FGFR signaling.^50^

### Limitations

This study had some limitations. As a single-institution hospital-based study, the characteristics of our patient population receiving NACT might not be representative of the general population (eg, higher proportion of White patients with TNBC, higher proportion of grade 3 disease). To address this limitation, we conducted a parallel analysis in the NCDB and observed consistent results using data from 109 082 patients.^34^ Another major limitation of the study is the small sample size of matched primary and residual tumor pairs. This limited our ability to detect somatic alterations that changed significantly posttreatment among different racial groups and subtypes. Therefore, the racial differences in MAPK and PI3K/AKT pathways observed in our study and its potential role in contributing to the racial disparity in response to NACT needs further confirmation from analysis using pretreatment and posttreatment tumors. The study also lacked detailed socioeconomic and germline data, which we hope to address in future studies.

## Conclusions

In this cohort study of patients with breast cancer receiving NACT, we found that achieving pCR was associated with improved long-term survival outcomes, with Black patients less likely to achieve pCR compared with their White counterparts in general. The racial disparity in pCR rate varied across different breast cancer subtypes and was most profound among patients with HR-/*ERBB2*+ disease. To better understand what might have contributed to the observed racial disparity, further analysis on the granular data revealed potential systematic barriers, such as treatment delays and observed potential resistance mechanisms such as differences in MAPK and PI3K/AKT pathways.

## Notes

Supplement 1. **eTable 1.** Characteristics of Patients With DNA Sequencing Results Based on Their Sample Type (i.e. Primary, Residual, Paired) **eTable 2.** Hazard Ratios for Different Tumor and Treatment Factors in Overall Survival and Recurrence-Free Survival **eTable 3.** Odds Ratio Between Black and White Patients in pCR Adjusted for Tumor and Treatment Characteristics Sequentially **eTable 4.** Odds Ratios of Achieving pCR Adjusted for Selected Demographic and Clinical Factors **eTable 5.** Most Common Treatment Regimens During Chemotherapy for HER2- and HER2+ Patients **eTable 6.** Molecular Characteristics of HR+/HER2- Patients **eTable 7.** Odds Ratios of Achieving pCR Adjusted for Selected Molecular Characteristics Among HR+/HER2- Patients **eTable 8.** Somatic Alterations With Significant Difference Between Primary and Residual Tumors in All Patients and Over 10% Difference Within Each Breast Cancer Subtype **eFigure 1.** Flow Chart of Patients Selected for the Analysis (Online Only) **eFigure 2.** Mutational Landscape of 13 Pairs of Primary and Residual Tumor Samples Stratified by Breast Cancer Subtypes (Online Only) **eFigure 3.** Mutational Landscape of Primary Tumor Samples From 141 Black and White Patients Stratified by Breast Cancer Subtypes (Online Only) **eFigure 4.** Mutational Landscape of Residual Tumor Samples From 42 Black and White Patients Stratified by Breast Cancer Subtypes (online only)

Supplement 2.
Data Sharing Statement
