# Supplementary material for: Racial Disparities in Pathological Complete Response Among Patients Receiving Neoadjuvant Chemotherapy for Early-Stage Breast Cancer
Source: JAMA Netw Open. 2023 Mar 30;6(3):e233329. doi: 10.1001/jamanetworkopen.2023.3329 (PMC10064259; doi:10.1001/jamanetworkopen.2023.3329)
Supplement: Supplement 1. — eTable 1. Characteristics of Patients With DNA Sequencing Results Based on Their Sample Type (i.e. Primary, Residual, Paired) eTable 2. Hazard Ratios for Different Tumor and Treatment Factors in Overall Survival and Recurrence-Free Survival eTable 3. Odds Ratio Between Black and White Patients in pCR Adjusted for Tumor and Treatment Characteristics Sequentially eTable 4. Odds Ratios of Achieving pCR Adjusted for Selected Demographic and Clinical Factors eTable 5. Most Common Treatment Regimens During Chemotherapy for HER2- and HER2+ Patients eTable 6. Molecular Characteristics of HR+/HER2- Patients eTable 7. Odds Ratios of Achieving pCR Adjusted for Selected Molecular Characteristics Among HR+/HER2- Patients eTable 8. Somatic Alterations With Significant Difference Between Primary and Residual Tumors in All Patients and Over 10% Difference Within Each Breast Cancer Subtype eFigure 1. Flow Chart of Patients Selected for the Analysis (Online Only) eFigure 2. Mutational Landscape of 13 Pairs of Primary and Residual Tumor Samples Stratified by Breast Cancer Subtypes (Online Only) eFigure 3. Mutational Landscape of Primary Tumor Samples From 141 Black and White Patients Stratified by Breast Cancer Subtypes (Online Only) eFigure 4. Mutational Landscape of Residual Tumor Samples From 42 Black and White Patients Stratified by Breast Cancer Subtypes (online only) [file jamanetwopen-e233329-s001.pdf]

Supplemental Online Content

Zhao F, Miyashita M, Hattori M, et al. Racial disparities in pathological complete response among patients receiving neoadjuvant chemotherapy for early-stage breast cancer. *JAMA Netw Open*. 2023;6(3):e233329.
doi:10.1001/jamanetworkopen.2023.3329

eTable 1. Characteristics of Patients With DNA Sequencing Results Based on Their Sample Type (i.e. Primary, Residual, Paired)

eTable 2. Hazard Ratios for Different Tumor and Treatment Factors in Overall Survival and Recurrence-Free Survival

eTable 3. Odds Ratio Between Black and White Patients in pCR Adjusted for Tumor and Treatment Characteristics Sequentially

eTable 4. Odds Ratios of Achieving pCR Adjusted for Selected Demographic and Clinical Factors

eTable 5. Most Common Treatment Regimens During Chemotherapy for HER2- and HER2+ Patients

eTable 6. Molecular Characteristics of HR+/HER2- Patients

eTable 7. Odds Ratios of Achieving pCR Adjusted for Selected Molecular Characteristics Among HR+/HER2- Patients

eTable 8. Somatic Alterations With Significant Difference Between Primary and Residual Tumors in All Patients and Over 10% Difference Within Each Breast Cancer Subtype

eFigure 1. Flow Chart of Patients Selected for the Analysis (Online Only)

eFigure 2. Mutational Landscape of 13 Pairs of Primary and Residual Tumor Samples Stratified by Breast Cancer Subtypes (Online Only)

eFigure 3. Mutational Landscape of Primary Tumor Samples From 141 Black and White Patients Stratified by Breast Cancer Subtypes (Online Only)

eFigure 4. Mutational Landscape of Residual Tumor Samples From 42 Black and White Patients Stratified by Breast Cancer Subtypes (online only)

This supplemental material has been provided by the authors to give readers additional information about their work.

© 2023 Zhao F et al. *JAMA Network Open.*

eTable 1. Characteristics of patients with DNA sequencing results based on their sample type (i.e. primary, residual, paired)

Factor, No. (%)	Primary (n = 140)	Residual (n = 33)	Paired (n = 13)	*P*-value [a]
Race/Ethnicity				.91
Black	64 (45.7)	16 (48.5)	8 (61.5)	
White	65 (46.4)	14 (42.4)	4 (30.8)	
Others [b]	11 (7.9)	3 (9.1)	1 (7.7)	
Received Neoadjuvant Therapy				< .001
No	132 (94.3)	0	0	
Yes	8 (5.7)	33 (100.0)	13 (100.0)	
Age at Diagnosis, mean (SD), years	53.7 (14.3)	50.6 (12 .6)	53.9 (17.1)	.25
Breast Cancer Subtype				.28
HR+/HER2-	59 (50.0)	14 (42.4)	4 (30.8)	
HR+/HER2+	16 (13.6)	9 (27.3)	5 (38.5)	
HR-/HER2+	9 (7.6)	3 (9.1)	1 (7.7)	
TNBC	34 (28.8)	7 (21.2)	3 (23.1)	
missing	22	0	0	
Tumor Grade				.044
1	11 (8.5)	0	0	
2	62 (47.7)	11 (34.4)	3 (23.1)	
3	57 (43.8)	21 (65.6)	10 (76.9)	
missing	10	1	0	
AJCC Stage				.004
I	40 (28.6)	1 (3.0)	0	
II	64 (45.7)	18 (54.5)	11 (84.6)	
III	35 (25.0)	12 (36.4)	2 (15.4)	
IV	1 (0.7)	2 (6.1)	0	
Recurrence				.23
No	93 (69.4)	22 (66.7)	9 (69.2)	
Local/Regional	9 (6.7)	0	0	
Distant	32 (23.9)	11 (33.3)	4 (30.8)	
missing	6	0	0	
Charlson Comorbidity Index				.39
0	111 (79.3)	25 (75.8)	10 (76.9)	
1	11 (7.9)	5 (15.2)	2 (15.4)	
≥ 2	18 (12.9)	3 (9.1)	1 (7.7)	

[a] p-values for the comparison between patients with primary samples and residual samples were estimated using t-tests for age at diagnosis; χ2 tests for race/ethnicity, tumor subtype (excluding missing categories) and Charlson comorbidity index; Fisher's exact tests for receipt of neoadjuvant therapy, tumor grade (excluding missing categories), tumor stage and recurrence (excluding missing categories).
[b] Others include 6 Asian patients and 5 Hispanic patients and 4 multiethnic/unknown.

© 2023 Zhao F et al. *JAMA Network Open.*

eTable 2. Hazard ratios for different tumor and treatment factors in overall survival and recurrence-free survival

	Overall Survival Hazard Ratio (95% CI) [a]	Recurrence-free Survival Hazard Ratio (95% CI) [b]
Race/ethnicity		
White	1 (ref.)	1 (ref.)
Black	2.37 (1.49 to 3.77)	1.75 (1.22 to 2.49)
Others [c]	1.99 (0.93 to 4.29)	1.59 (0.88 to 2.88)
Age at diagnosis, every 10 years	1.14 (0.98 to 1.33)	1.08 (0.95 to 1.22)
pCR		
Yes	1 (ref.)	1 (ref.)
No	6.10 (2.80 to 13.32)	5.54 (3.10 to 9.88)
Subtype		
HR+/HER2-	1 (ref.)	1 (ref.)
HR+/HER2+	0.71 (0.35 to 1.42)	0.90 (0.53 to 1.52)
HR-/HER2+	1.87 (0.98 to 3.57)	1.43 (0.80 to 2.55)
TNBC	1.43 (0.90 to 2.29)	1.65 (1.12 to 2.43)
AJCC Stage		
I	1 (ref.)	1 (ref.)
II	1.50 (0.59 to 3.79)	1.19 (0.64 to 2.21)
III	3.71 (1.45 to 9.48)	2.34 (1.24 to 4.41)
Charlson Comorbidity Index		
0	1 (ref.)	
1	1.49 (0.77 to 2.86)	
≥ 2	1.93 (1.15 to 3.23)	

[a] Hazard ratio for overall survival adjusted for race/ethnicity, age, pCR, tumor subtype, tumor stage and comorbidity index in Cox proportional-hazards model
[b] Hazard ratio for recurrence-free survival adjusted for race/ethnicity, age, pCR, tumor subtype and tumor stage in Cox proportional hazards model
[c] Other patients include 35 Asian patients, 30 Hispanic patients and 1 Native American patient

© 2023 Zhao F et al. *JAMA Network Open.*

eTable 3. Odds ratio between Black and White patients in pCR adjusted for tumor and treatment characteristics sequentially

Variables adjusted in model	Odds Ratio for Black vs. White patients (95% CI)
Unadjusted	0.69 (0.49 to 0.98)
Adjusted for age at diagnosis	0.73 (0.51 to 1.03)
+ subtype	0.68 (0.47 to 0.97)
+ grade	0.66 (0.46 to 0.95)
+ clinical T stage	0.70 (0.49 to 1.01)
+ clinical N stage	0.71 (0.49 to 1.03)
+ delay in chemotherapy	0.78 (0.54 to 1.14)
+ duration of chemotherapy	0.72 (0.49 to 1.06)

© 2023 Zhao F et al. *JAMA Network Open.*

eTable 4. Odds ratios of achieving pCR adjusted for selected demographic and clinical factors

	No. of patients	No. of pCR	% of pCR	Odds Ratio (95% CI) [a]
Race/ethnicity				
White	355	130	36.6	1 (ref.)
Black	269	77	28.6	0.72 (0.49 to 1.06)
Others [b]	66	22	33.3	0.79 (0.43 to 1.45)
Age at diagnosis, per 10 years increase	690	229	33.2	0.85 (0.74 to 0.98)
Clinical T-stage, per unit increase	690	229	33.2	0.70 (0.55 to 0.89)
Clinical N-stage				
N0	318	128	40.3	1 (ref.)
≥ N1	372	101	27.2	0.58 (0.40 to 0.83)
Subtype				
HR+/HER2-	224	44	19.6	1 (ref.)
HR+/HER2+	141	48	34.0	1.86 (1.11 to 3.10)
HR-/HER2+	83	48	57.8	5.52 (3.08 to 9.90)
TNBC	242	89	36.8	1.79 (1.13 to 2.85)
Tumor grade				
1	14	5	35.7	1.37 (0.41 to 4.58)
2	146	31	21.2	0.51 (0.31 to 0.83)
3	502	182	36.3	1 (ref.)
Delay in chemotherapy, weeks				
≤ 4	228	95	38.3	1 (ref.)
4 - 8	338	112	33.1	0.74 (0.51 to 1.08)
> 8	104	22	21.2	0.34 (0.19 to 0.62)
Duration of chemotherapy, weeks				
≤ 15	176	47	26.7	1 (ref.)
15 - 20	252	90	35.7	1.75 (1.11 to 2.76)
> 20	96	37	38.5	1.97 (1.09 to 3.55)
Year of diagnosis [c]				
2002 - 2010	140	38	27.1	1 (ref.)
2011 - 2015	278	91	32.7	1.29 (0.78 to 2.13)
2016 - 2020	272	100	36.8	1.47 (0.88 to 2.44)
Body Mass Index (BMI), kg/m^2				
Underweight (< 18.5)	5	1	20.0	0.29 (0.03 to 3.24)
Normal (18.5 – 24.9)	200	68	34.0	1 (ref.)
Overweight (25 – 29.9)	174	62	35.6	1.34 (0.84 to 2.16)
Obese (30 – 34.9)	116	40	34.5	1.54 (0.89 to 2.67)
Severely Obese (≥ 35)	95	32	33.7	1.73 (0.94 to 3.18)

[a] Odds ratio of achieving pCR adjusted for demographic and clinical factors using multivariate logistic regression, missing categories are not shown in the table

[b] Other patients include 35 Asian patients, 30 Hispanic patients and 1 Native American patient

[c] Year of diagnosis and BMI were not included in the final multivariate model, the odds ratios of achieving pCR for these two variables were further adjusted in the multivariate model including all the variables listed above them

© 2023 Zhao F et al. *JAMA Network Open.*

eTable 5. Most common treatment regimens during chemotherapy for HER2- and HER2+ patients

	**HER2- breast cancer patients**		
	Black patients (n = 182)	White patients (n = 242)	*P*-value[h]
Treatment Regimen, No. (%)			.13
AC-T or TAC[a]	145 (79.7)	185 (76.8)	
AC[b]	2 (1.1)	11 (4.6)	
TC[c]	9 (4.9)	16 (6.6)	
T-Carbo[d]	6 (3.3)	8 (3.3)	
Paclitaxel only	9 (4.9)	6 (2.5)	
Pembrolizumab + chemotherapy	4 (2.2)	11 (4.6)	
Others	7 (3.9)	4 (1.6)	
missing	0	1	
	**HER2+ breast cancer patients**		
	Black patients (n = 87)	White patients (n = 113)	*P*-value
Treatment Regimen, No. (%)			.14
TCHP or TCH[e]	42 (48.8)	69 (61.6)	
ACTHP or ACTH[f]	25 (29.1)	31 (27.7)	
THP or TH[g]	14 (16.3)	9 (8.0)	
Others	5 (5.8)	3 (2.7)	
missing	1	1	

[a] AC-T (doxorubicin and cyclophosphamide followed by paclitaxel or docetaxel); TAC (doxorubicin, cyclophosphamide and docetaxel)
[b] doxorubicin and cyclophosphamide
[c] cyclophosphamide and docetaxel
[d] paclitaxel/docetaxel and carboplatin
[e] TCHP (docetaxel, carboplatin, trastuzumab and pertuzumab); TCH (docetaxel, carboplatin and trastuzumab)
[f] ACTHP (doxorubicin and cyclophosphamide followed by paclitaxel, trastuzumab and pertuzumab); ACTH (doxorubicin and cyclophosphamide followed by paclitaxel and trastuzumab)
[g] THP (paclitaxel, trastuzumab and pertuzumab); TH (paclitaxel and trastuzumab)
[h] p-values for the comparison between White patients and Black patients were estimated using Fisher's exact tests (excluding missing categories)

© 2023 Zhao F et al. *JAMA Network Open.*

eTable 6. Molecular characteristics of HR+/HER2- patients

Factor, No. (%)	Black Patients (n = 78)	White Patients (n = 126)	Others [a] (n = 20)	*P*-value [b]
Intrinsic Subtype Surrogate [c]				0.081
Luminal A	18 (25.7)	36 (38.7)	9 (50.0)	
Luminal B	52 (74.3)	57 (61.3)	9 (50.0)	
missing	8	33	2	
ER H-score, median (IQR)	160 (20, 270)	180 (57.5, 270)	150 (20, 277.5)	0.16
ER H-score				0.14
< 50	21 (34.4)	18 (22.8)	5 (31.3)	
50 - 250	20 (32.8)	28 (35.4)	4 (25.0)	
≥ 250	20 (32.8)	33 (41.8)	7 (43.8)	
missing	17	47	4	
PR H-score, median (IQR)	2 (0, 106.7)	60 (0, 155)	30 (0, 225)	0.086
PR H-score				0.017
< 50	39 (68.4)	35 (45.5)	8 (53.3)	
50 -250	13 (22.8)	34 (44.2)	5 (33.3)	
≥ 250	5 (8.8)	8 (10.4)	2 (13.3)	
missing	21	49	5	

[a] Other patients include 8 Asian patients and12 Hispanic patients
[b] p-values for the comparison between Black and White patients were estimated using χ2 tests for intrinsic subtype surrogate and Wilcoxon rank-sum tests for ER and PR H scores (all excluding missing categories)
[c] HR+/HER2- patients were categorized into luminal A-like and luminal B-like based on the current ESMO guideline: luminal A [ER+, HER2-, PR high (≥ 20%) and Ki67 clearly low (≤ 10%)] and luminal B [ER+, HER2-, and either PR low (< 20%) or Ki67 clearly high (≥ 30%)]. If patients had Ki67 missing or between 10-30%, the categorization was based on PR percentage only.

© 2023 Zhao F et al. *JAMA Network Open.*

eTable 7. Odds ratios of achieving pCR adjusted for selected molecular characteristics among HR+/HER2- patients

	No. of patients	No. of pCR	% of pCR	Odds Ratio (95% CI) [a]
Intrinsic Subtype Surrogate				
Luminal A	63	7	11.1	1 (ref.)
Luminal B	118	33	28.0	3.15 (1.25 to 7.88)
ER H-score, per 10 unit decrease				1.07 (1.03 to 1.12)
ER H-score				
< 50	44	18	40.9	1 (ref.)
50 - 250	52	10	19.2	0.40 (0.15 to 1.04)
≥ 250	60	6	10.0	0.23 (0.08 to 0.67)
PR H-score, per 10 unit decrease				1.08 (1.02 to 1.14)
PR H-score				
< 50	82	26	31.7	1 (ref.)
50 -250	52	6	11.5	0.33 (0.12 to 0.92)
≥ 250	15	1	6.7	0.17 (0.02 to 1.41)

[a] Odds ratio of achieving pCR for each variable in multivariate logistic regression in addition to adjusting for racial/ethnic groups, age at diagnosis, clinical T- and N-stage and delay in treatment, missing categories are not shown in the table

© 2023 Zhao F et al. *JAMA Network Open.*

eTable 8. Somatic alterations with significant difference between primary and residual tumors in all patients and over 10% difference within each breast cancer subtype

Gene [a]	No. of patients with mutation/ No. of patients (%)		*P*-value [b]	Benjamini-Hochberg adjusted *P*-value [c]	Oncogenic Signaling Pathway [d]
	Primary	Residual			
Total [e]					
FGF4	3/153 (2.0)	6/46 (13.0)	.016	.031	
FAT1	21/153 (13.7)	0/46	.020	.031	Hippo
FGF3	2/153 (1.3)	5/46 (10.9)	.026	.031	
*CCND1**	8/153 (5.2)	8/46 (17.4)	.027	.031	Cell cycle
*MCL1**	47/153 (30.7)	5/46 (10.9)	.031	.031	
HR+/HER2-					
*CCND1**	7/63 (11.1)	6/18 (33.3)	.034	.16	Cell cycle
FGF4	3/63 (4.8)	4/18 (22.2)	.040	.16	
*MCL1**	18/63 (28.6)	1/18 (5.6)	.057	.16	
*GATA3**	6/63 (9.5)	5/18 (27.8)	.060	.16	
*MAP3K1**	17/63 (27)	1/18 (5.6)	.061	.16	
FGF19	4/63 (6.3)	4/18 (22.2)	.068	.16	
FGF3	2/63 (3.2)	3/18 (16.7)	.070	.16	
*PIK3CA**	21/63 (33.3)	10/18 (55.6)	.10	.19	PI-3-Kinase/Akt
FAT1	10/63 (15.9)	0/18	.11	.19	Hippo
BCORL1	8/63 (12.7)	0/18	.19	.26	
SMARCA4	8/63 (12.7)	0/18	.19	.26	
ZFHX3	9/63 (14.3)	0/18	.20	.26	
*CDH1**	12/63 (19)	6/18 (33.3)	.21	.26	
*TP53**	27/63 (42.9)	5/18 (27.8)	.29	.33	TP53
ERCC3	7/63 (11.1)	0/18	.34	.34	
GATA1	7/63 (11.1)	0/18	.34	.34	
HR+/HER2+ and HR-/HER2+					
ERCC3	0/31	3/18 (16.7)	.044	.20	
ATM	6/31 (19.4)	0/18	.073	.20	TP53
FLCN	6/31 (19.4)	0/18	.073	.20	
*PIK3CA**	13/31 (41.9)	3/18 (16.7)	.11	.20	PI-3-Kinase/Akt
TOP2A	8/31 (25.8)	1/18 (5.6)	.13	.20	
BCL6	0/31	2/18 (11.1)	.13	.20	
*CCND1**	0/31	2/18 (11.1)	.13	.20	Cell cycle
FGF3	0/31	2/18 (11.1)	.13	.20	
FGF4	0/31	2/18 (11.1)	.13	.20	

© 2023 Zhao F et al. *JAMA Network Open.*

SMARCB1	0/31	2/18 (11.1)	.13	.20	
*ERBB2**	22/31 (71.0)	9/18 (50.0)	.22	.30	RTK-RAS
CKS1B	4/31 (12.9)	0/18	.28	.31	
*RB1**	4/31 (12.9)	0/18	.28	.31	Cell cycle
RARA	8/31 (25.8)	2/18 (11.1)	.29	.31	
CDK12	12/31 (38.7)	5/18 (27.8)	.54	.54	
TNBC					
*PTEN**	1/37 (2.7)	3/10 (30.0)	.026	.33	PI-3-Kinase/Akt
*CCNE1**	2/37 (5.4)	3/10 (30.0)	.057	.33	Cell cycle
CIC	1/37 (2.7)	2/10 (20.0)	.11	.33	
*MCL1**	9/37 (24.3)	0/10	.17	.33	
KMT2D	2/37 (5.4)	2/10 (20.0)	.19	.33	
INPP4B	0/37	1/10 (10.0)	.21	.33	PI-3-Kinase/Akt
LRP1B	0/37	1/10 (10.0)	.21	.33	
SDHA	0/37	1/10 (10.0)	.21	.33	
FGFR2	0/37	1/10 (10.0)	.21	.33	RTK-RAS
SGK1	0/37	1/10 (10.0)	.21	.33	
GNA13	0/37	1/10 (10.0)	.21	.33	
AKT3	0/37	1/10 (10.0)	.21	.33	PI-3-Kinase/Akt
TRAF3	0/37	1/10 (10.0)	.21	.33	
*SPEN**	0/37	1/10 (10.0)	.21	.33	Notch
GRIN2A	0/37	1/10 (10.0)	.21	.33	
*FBXW7**	0/37	1/10 (10.0)	.21	.33	Notch
HRAS	0/37	1/10 (10.0)	.21	.33	RTK-RAS
NOTCH1	6/37 (16.2)	0/10	.32	.46	Notch
*CDKN1B**	4/37 (10.8)	0/10	.56	.56	Cell cycle
*PIK3CA**	4/37 (10.8)	0/10	.56	.56	PI-3-Kinase/Akt
*MYC**	4/37 (10.8)	0/10	.56	.56	Myc
DNMT3A	4/37 (10.8)	0/10	.56	.56	
ETV6	4/37 (10.8)	0/10	.56	.56	
CDK4	4/37 (10.8)	0/10	.56	.56	Cell cycle
CKS1B	4/37 (10.8)	0/10	.56	.56	
*BRCA1**	4/37 (10.8)	0/10	.56	.56	

[a] Genes that were previously reported as breast cancer driver genes were marked with*.
[b] p-values were estimated using exact logistic regression for all samples adjusting for subtype (including a missing category) and Fisher's exact tests for the subtype stratified analysis.
[c] adjusted p-values were estimated using Benjamini-Hochberg adjustments.
[d] Somatic alterations were characterized into ten canonical pathways: cell cycle, Hippo, Myc, Notch, Nrf2, PI-3-Kinase/Akt, RTK-RAS, TGFβ signaling, p53 and β-catenin/Wnt.
[e] 22 patients missing subtype information were also included in the pooled analysis.

© 2023 Zhao F et al. *JAMA Network Open.*

eFigure 1. Flow chart of patients selected for the analysis (online only)

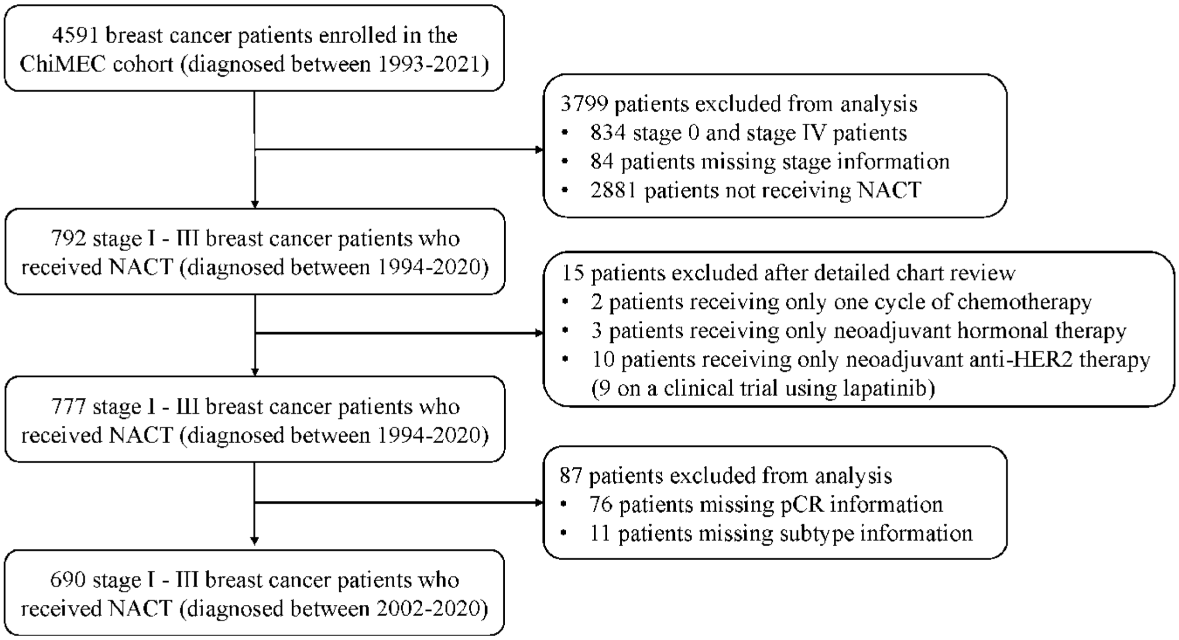

© 2023 Zhao F et al. *JAMA Network Open.*

eFigure 2. Mutational landscape of 13 pairs of primary and residual tumor samples stratified by breast cancer subtypes (online only)

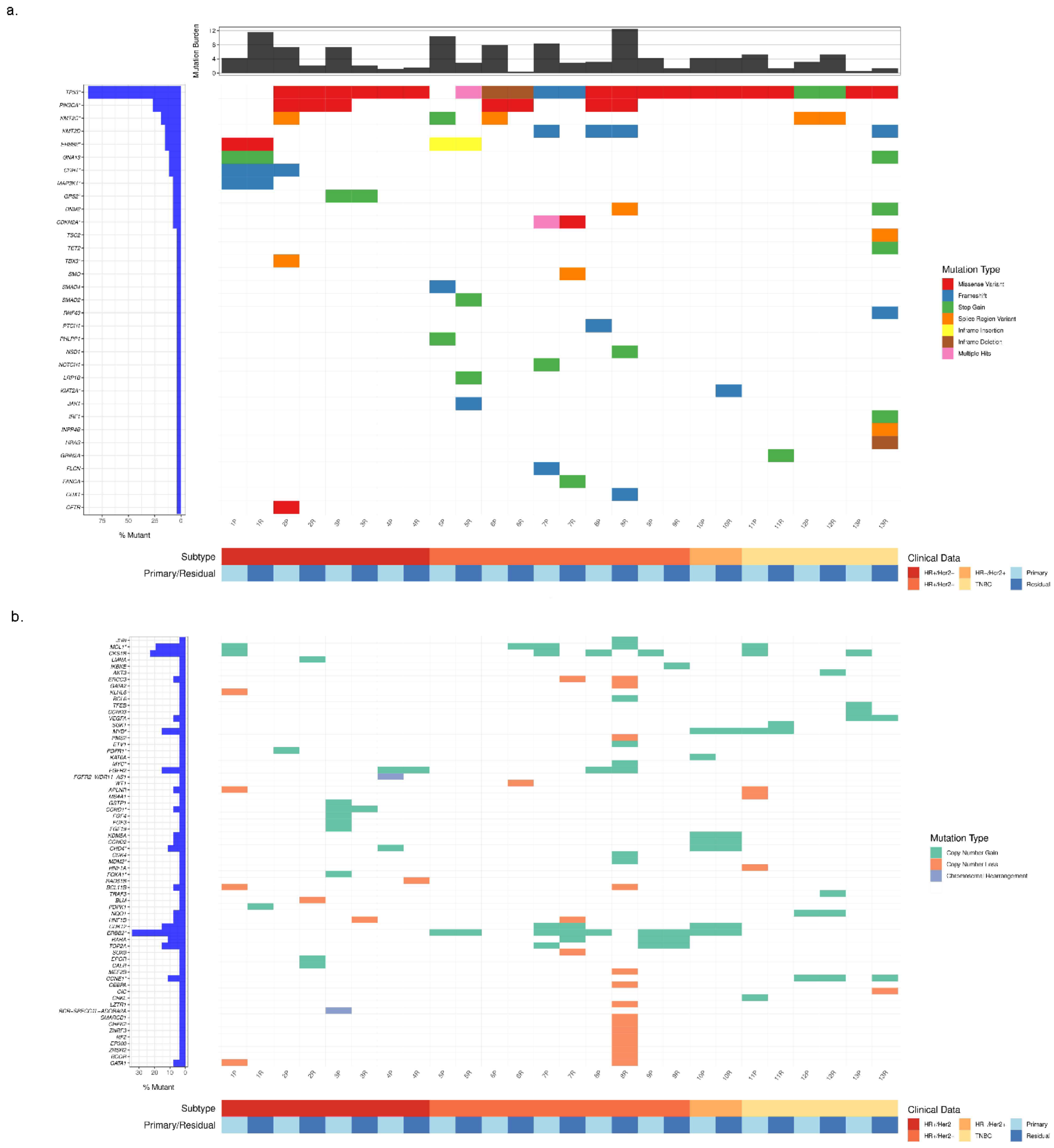

e2a. Oncoprint describing the genes that had point mutations observed with the 13 paired samples listed side by side. Genes were ordered by decreasing frequency; e2b. Oncoprint describing the genes with copy number or chromosomal variation observed between the 13 paired samples. Genes were ordered by their locations. Genes that were previously reported as breast cancer driver genes were marked with*.

© 2023 Zhao F et al. *JAMA Network Open.*

eFigure 3. Mutational landscape of primary tumor samples from 141 Black and White patients stratified by breast cancer subtypes (online only)

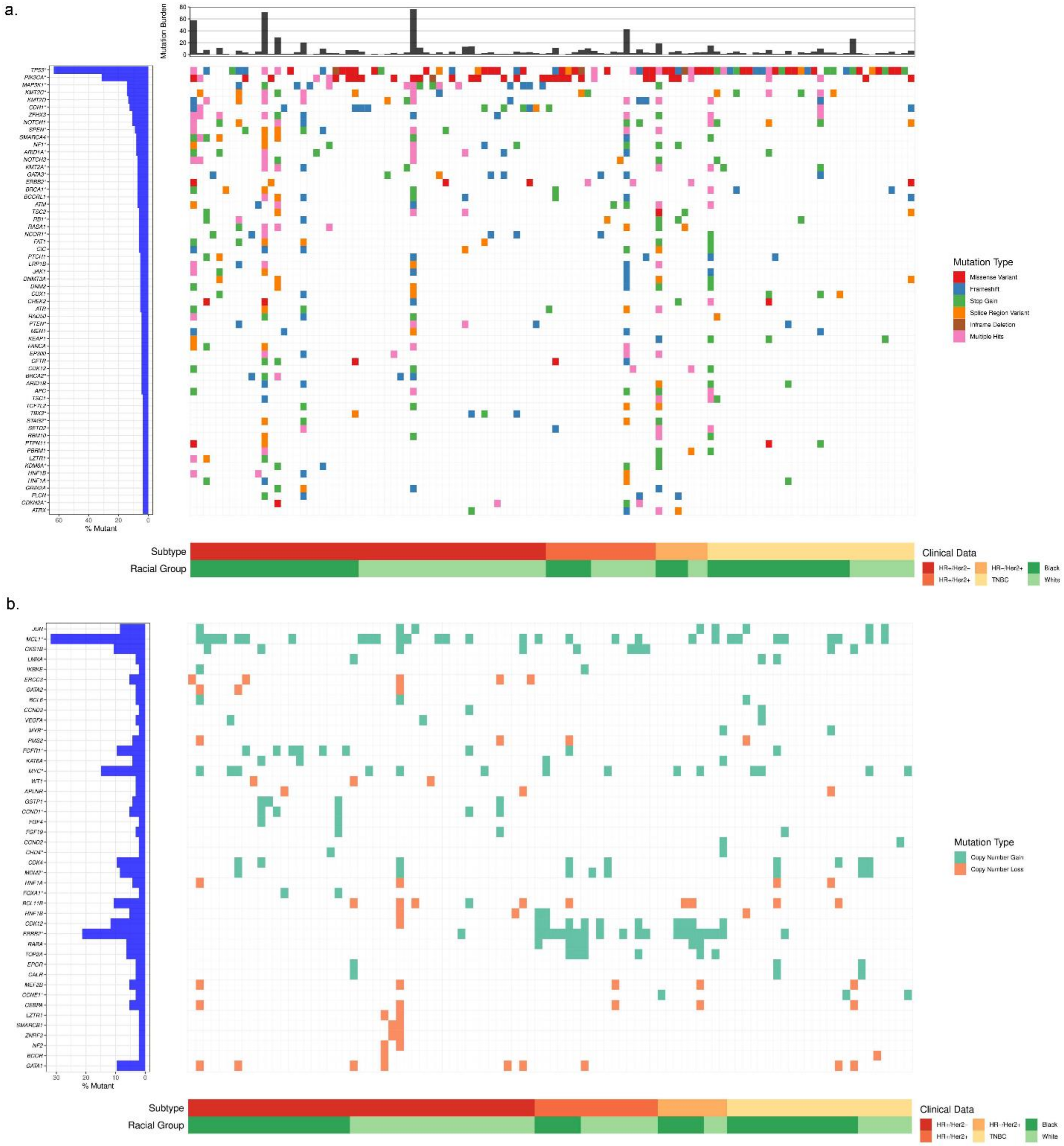

e3a. Oncoprint describing the genes that had point mutations observed for at least 3% of the samples. Genes were ordered by decreasing frequency; e3b. Oncoprint describing the genes with copy number or chromosomal variation detected for at least 2% of the samples. Genes were ordered by their locations. Genes that were previously reported as breast cancer driver genes were marked with*. Samples were not plotted if no alterations were observed for the listed genes and/or the subtype data was missing.

© 2023 Zhao F et al. *JAMA Network Open.*

eFigure 4. Mutational landscape of residual tumor samples from 42 Black and White patients stratified by breast cancer subtypes (online only)

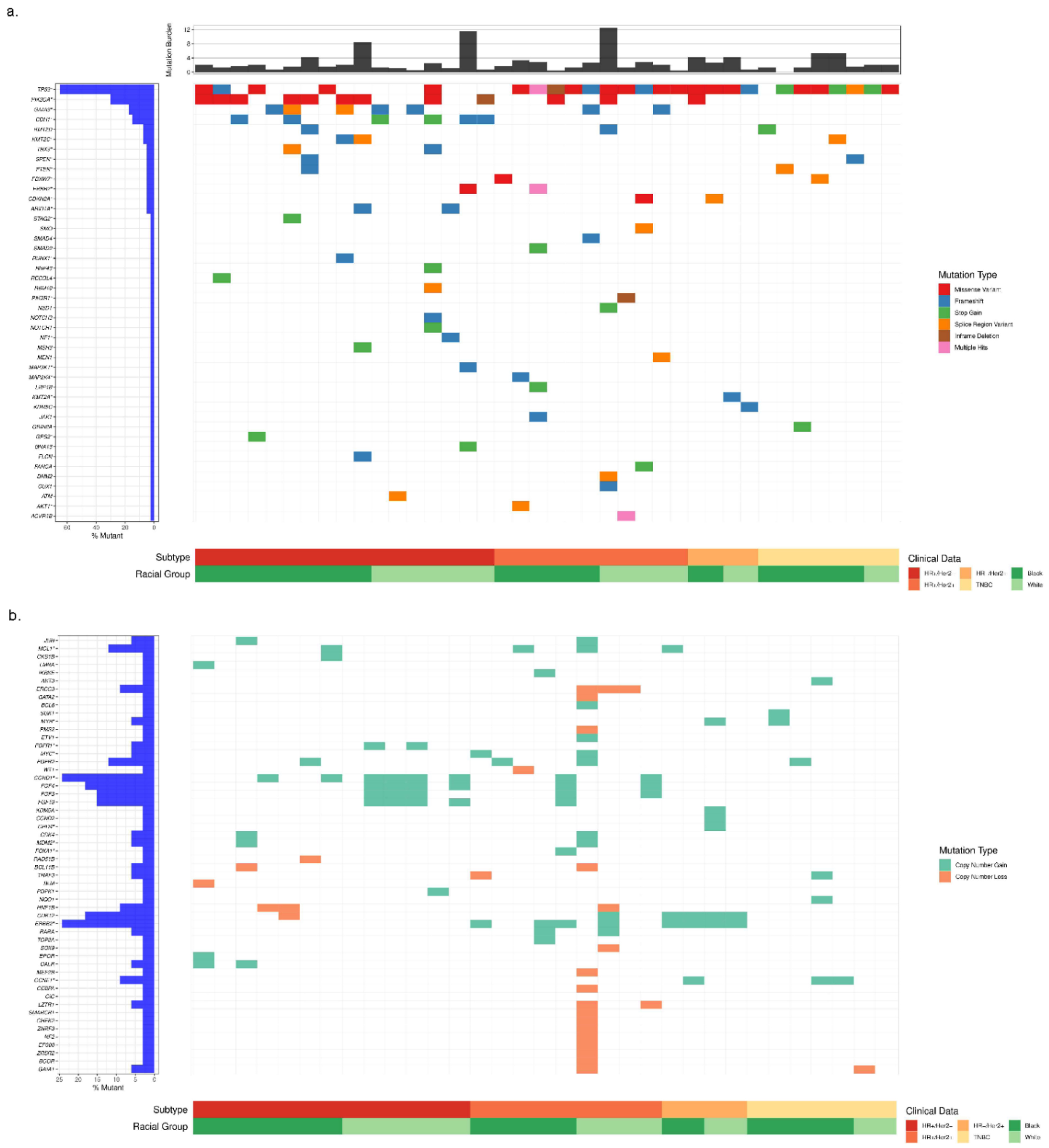

e4a. Oncoprint describing the genes that had point mutations observed for at least 2% of the samples. Genes were ordered by decreasing frequency; e4b. Oncoprint describing the genes with copy number or chromosomal variation detected for at least 2% of the samples. Genes were ordered by their locations. Genes that were previously reported as breast cancer driver genes were marked with*. Samples were not plotted if no alterations were observed for the listed genes.

© 2023 Zhao F et al. *JAMA Network Open.*
