# Supplementary material for: Racial Disparities in Pathological Complete Response Among Patients Receiving Neoadjuvant Chemotherapy for Early-Stage Breast Cancer
Source: JAMA Netw Open. 2023 Mar 30;6(3):e233329. doi: 10.1001/jamanetworkopen.2023.3329 (PMC10064259; doi:10.1001/jamanetworkopen.2023.3329)
Supplement: Supplement 2. — Data Sharing Statement [file jamanetwopen-e233329-s002.pdf]

Data Sharing Statement

Zhao. Racial Disparities in Pathological Complete Response Among Patients Receiving Neoadjuvant Chemotherapy for Early-Stage Breast Cancer. *JAMA Netw Open*. Published March 30, 2023. doi:10.1001/jamanetworkopen.2023.3329

Data
Data available: No
